# Cold storage effects on lethal and sublethal responses of *Amphibalanus amphitrite* Nauplii

**DOI:** 10.1007/s10646-022-02571-1

**Published:** 2022-07-15

**Authors:** Veronica Piazza, Chiara Gambardella, Elisa Costa, Roberta Miroglio, Marco Faimali, Francesca Garaventa

**Affiliations:** National Research Council, Institute for the Study of Anthropic Impact and Sustainability in the marine environment (CNR-IAS), via de Marini 16, 16140 Genova, Italy

**Keywords:** *Amphibalanus amphitrite*, Bioassays, Immobilization, Marine invertebrates, Mortality, Swimming

## Abstract

Bioassays are extensively used in ecotoxicology and there is a constant need for even more sensitive, reliable and easy to rear and obtain model organisms. Larvae of the crustacean *Amphibalanus amphitrite* are a good ecotoxicological model, for their high sensitivity to a wide range of toxicants and emerging contaminants. A standardized protocol for this toxicity bioassay has been recently proposed. Nevertheless, a limit of this model organism is the lack of resting stages and the need to use larvae immediately after their release from adults, thus increasing laboratory efforts related to the maintenance of adults. The aim of this work is to verify if short-term cold storage of *A. amphitrite* larvae prior to use in ecotoxicological tests may affect the ecotoxicological responses of these organisms. Three end-points (mortality, immobilization and swimming speed alteration) were measured on nauplii after storing them at 4 ± 1 °C for different times (24, 72 and 120 h) before bioassay set-up. Bioassays were set up using: (i) clean filtered natural sea water (0.22 µm FNSW), (ii) a reference toxicant (Cadmium Nitrate) and (iii) an environmental matrix (sediment elutriate). Results show that mortality, differently from the other two endpoints, was not affected by cold-storage. Even after 5 days of larvae storage at 4 ± 1 °C before bioassay set up, mortality data were comparable to those obtained for non-cold-stored organisms. Moreover, larval sensitivity to the reference toxicant and sediment elutriate did not change. Regarding the other two end points, low cadmium concentrations significantly changed immobility and swimming activity in cold-stored nauplii compared to larvae used immediately after larval release. In conclusion, short-term cold storage of *A. amphitrite* nauplii before bioassay set up is an appropriate procedure in ecotoxicological testing if mortality is the endpoint to be considered for final evaluation.

## Introduction

Aquatic ecotoxicology focuses on the interactions between aquatic organisms and pollutants, in order to evaluate related hazards, for both environmental and human health. A review of the most important organisms used in aquatic toxicology has been proposed by Nikinmaa (2014), discussing the benefits and arising issues related to the use of different model species. Commonly, risk assessments are performed by using algae (primary producers), invertebrates (primary consumers) and fish (secondary consumers) as model organisms. There is an increased need to expand the number of model species in ecotoxicology, also because of their selective toxicity towards contaminants. For instance, crustaceans, having a cephalic nervous system, have been found to be more sensitive than other invertebrates (such as echinoderms or bivalves) to insecticides (Bellas et al. 2005; His et al. 2000), polycyclic aromatic hydrocarbons (PAHs, Bellas and Thor 2007), chemicals (Marino-Balsa et al. 2000), and urban waste (de-la-Ossa-Carretero et al. 2012).

The use of aquatic invertebrates in environmental risk assessment has increased in the last few decades. Bioassays on aquatic invertebrates are more advisable than those on fish, due to ethical advantages (Van den Berg et al. 2020). Invertebrate larvae, such as early life stages of the crustacean *Artemia* sp., the rotifer *Brachionus plicatilis* or the sea-urchin *Paracentrotus lividus*, are model organisms commonly used in ecotoxicological assays (Bellas et al. 2005; Dahms et al. 2011; Fichet et al. 1998; Libralato et al. 2016; Manfra et al. 2015; van Dam et al. 2016).

Model organisms are selected on the basis of their characteristics, such as sensitivity, optimum handling in laboratory conditions, and easy availability/rearing. Indeed, organisms should be obtained in a short time and on-demand. For this reason, the most common model organisms proposed in marine water ecotoxicity kits should have resting stages (cysts) that can be easily reactivated before bioassay set up (i.e., the crustacean *Artemia salina* or the rotifer *Brachionus plicatilis*; Dahms et al. 2011; Libralato et al. 2016; Nunes et al. 2006). Otherwise, ecotoxicology laboratories need to make great efforts to constantly maintain adults in culture, even when they are needed for infrequent bioassays.

Larvae of the cirriped crustacean *Amphibalanus amphitrite* are a good ecotoxicological model. Barnacle adults can be easily held in laboratory culture, and they produce larvae (nauplii) throughout the year. This species has proved to be more sensitive than other organisms to a high number of toxicants (Rittschof et al. 1992; Sasikumar et al. 1995; Faimali et al. 2006a; Greco et al. 2006). A standardized bioassay protocol with *A. amphitrite* larvae is available (NU 2245/ 2012; Piazza et al. 2012). Moreover, the acute toxicity bioassay with *A. amphitrite* larvae has been recently introduced by the Italian legislation regarding dredging sediment management (D.M. 173/2016), thus raising interest in this species. However, a limit of this model organism is the lack of resting stages and the need to use larvae for bioassay set up within 4–6 h from their emission from adults (II stage nauplii), as stated in the standardized protocol.

Maintaining adult cultures (mollusks, echinoderms or crustaceans, such as *A. amphitrite*) is quite an expensive task that not all research groups can afford, thus limiting the possibility of performing thorough ecotoxicological assessments. The availability of high-quality biological material all year round, regardless of a species seasonal reproductive cycle, is one of the main constraints. Cryopreservation of gametes, embryos, and larvae of marine invertebrates could be a solution, thus making biological material available throughout the year, and enhancing the potential of such bioassays. Cryopreservation of marine invertebrates has been proposed for several development stages and cell types (i.e., sperm, oocytes, embryos and larvae) from a wide number of different species (Guo and Weng 2020), with a high commercial and ecological value. Specific cryopreservation protocols have been developed for marine vertebrates and invertebrates (Paredes 2015; 2019; Asturiano et al. 2017), mainly on species of global economic importance, including fish, sea urchins, and mollusks used in aquaculture (Campos et al. 2021; Cantarino Ribeiro et al. 2018). As to crustaceans, few studies have been proposed for larvae cryopreservation, for example for penaeid prawn larvae (Arun and Subramoniam 1997; Subramoniam and Arun 1999) and barnacles larvae, including *Balanus improvisus* and *A. amphitrite* (Gakhova et al. 1990; Khin-Maung-Oo et al. 1998). However, cryopreservation may be very aggressive at an ultrastructural level during freezing and thawing, and once organisms survive post-thaw, severe damage can occur (Gwo et al. 2003; Odintsova et al. 2009; Suquet et al. 2014), thus limiting their potential use in bioassays. Therefore, low temperature could represent a key biotic factor to be investigated to assess physiological changes in aquatic invertebrates (Van Dooremalen and Ellers 2010; Lee et al. 2018), before proposing cryopreservation prior to ecotoxicological surveys. In order to optimize laboratory efforts for the maintenance of adult cultures, and with a view to reducing required laboratory work to obtain newly hatched larvae as well as to supply larvae also to other laboratories. In this work we verify whether short-term cold storage (at 4 ± 1 °C) of *A. amphitrite* nauplii is an adequate procedure to keep larvae suitable to be used in bioassays. The aim is to highlight, through a multi end-point ecotoxicological test, any changes in organism responses related to larvae cold storage before bioassay set up. Three end-points with different sensitivity levels (mortality, immobilization, swimming speed alteration) were measured on nauplii after storage at 4 ± 1 °C for different times (24, 72 and 120 h) before bioassay set up. The experimental design featured three phases, each one considering any potential cold-storage effects on naupliar responses with and without the presence of a reference toxicant (Cadmium nitrate) or an environmental matrix (sediment elutriate), respectively.

Any recorded response changes of larvae after different cold storage times were analysed to determine cold storage times and end-points that were not affected by low-temperature conservation of larvae before bioassay set up.

## Materials and methods

### Model organism

*Amphibalanus (=Balanus) amphitrite*, (Darwin, 1854) is a crustacean with an adult sessile stage and a series of planktonic larval phases. Embryos, brooded in the mantle cavity of the adult organism, are released as II stage nauplii. Development goes through four naupliar planktotrophic stages (instars III-VI) and a final lecitotrophic stage, called cyprids. This latter stage, after a period of substratum exploration, produces a series of reversible connections (attachment phase) and then, after an irreversible fixation to the substratum (settlement phase), undergoes metamorphosis into the juvenile (Rittschof et al. 1992). This species has all the ideal characteristics to be proposed as an excellent model organism for ecotoxicity tests (Greco et al. 2006; Piazza et al. 2009,2012): wide geographic distribution, ecological relevance, easy to rear (its life cycle can be easily reproduced under laboratory conditions), relatively fast larval development, and a high sensitivity to a wide range of toxicants, including emerging contaminants.

### Reference toxicant

Cadmium Nitrate was selected as reference toxicant. Cd(NO_3_)_2_ was purchased from sigma Aldrich (St. Louis, MO, USA). It is commercially available in a 2% HNO_3_ standard solution for AA at 1000 ppm concentration. Stock solutions of Cadmium Nitrate at 100 ppm were prepared in 0.22 µm FNSW (Filtered Natural Sea Water) (Piazza et al. 2012) at the following concentrations: 0; 0.2; 0.4; 0.8; 1.6; 3.2 mg/L.

### Sediment eluate

Eluate was prepared according to EPA (2001). Seawater (0.22 µm Filtered Natural Sea Water 37‰ salinity, FNSW) was added to sediment in a 1:4 (w/v) ratio. The slurry was shaken at 240 rpm for 1 h in the dark and then centrifuged at 1200 *×* *g* for 15 min at 4 °C. The supernatant was then filtered through a 0.2 µm filter and then collected as eluate (100% eluate). The eluate was stored at 4 °C in the dark prior to toxicity testing conducted within 24 h, otherwise stored at −20 °C.

### Culture of barnacle larvae

II stage nauplii were obtained from laboratory cultures of brood stock of *A. amphitrite*. Twenty to thirty adult barnacles were reared in 800 ml beakers containing aerated Filtered (0.45 µm) Natural Sea Water (FNSW, 37‰) at 20 ± 1 °C, with a 16:8 h light: dark cycle. They were fed every two days with nauplii of *Artemia* sp. (100 mL, 20–35 larvae/mL), and *Tetraselmis suecica* (100 mL, 2 × 10^5^ cells/mL). Twenty beakers containing adults reared under the mentioned conditions produced nauplii throughout the year. Nauplii were collected with a 5 mL pipette by positioning the beaker near a light source. After collection, nauplii were filtered with a 80 µm net and transferred into a 500 ml beaker containing 0.22 µm FNSW at a density of 15–20 larvae/mL.

### Phase I: Preliminary bioassays with clean natural sea water

For non-cold storage tests (hereafter referred to as T0), 15–20 II stage nauplii of *A. amphitrite* (see 2.4) were transferred into multiwell plates, immediately after being collected from adults, each well containing 1 ml of clean 0.22 µm FNSW and stored at 20 °C in the dark. For cold storage trials, after collection, nauplii were filtered in 200 ml of clean 0.22 µm FNSW and stored at 4 ± 1 °C for 24, 72 and 120 h (hereafter named T1, T2, T3, respectively) in the dark. Cold storage of II stage nauplii was carried out by keeping larvae in a refrigerator with temperature set at 4 ± 1 °C (the control of temperature was ensured by a temperature data logger). Nauplii were not fed during all the storage period (from T1 to T3). After the different cold-storage times (T1, T2, T3), batches of 15–20 organisms were transferred into multiwell plates, each well containing in 1 ml of clean 0.22 µm FNSW and stored at 20 °C in the dark. According to the standardized protocol (NU 2245/2012), the end-point must be recorded after 24 h, by counting the number of dead and immobile larvae using a stereomicroscope. In this phase, the end-points were also recorded after 2 and 4 h from assay set up. The number of immobile organisms is composed of the amount of dead larvae (considering dead those larvae that do not swim and do not move any appendages for 10 s of observation) and of “non-swimming” larvae (considering “non-swimming” those larvae that do not shift their barycentre but move their appendages). Six replicates for each cold storage time were prepared; mortality and immobility percentages were calculated as mean value of the six replicates (M ± ES; *n* = 6).

In parallel, on the same larval batch, a behavioural end-point (Swimming Speed Alteration, SSA) was recorded according to Faimali et al. (2006a). Briefly, naupliar swimming speed was recorded in multiwell polystyrene plates after 2, 4 and 24 incubation hours at 20 °C from assay set up (as described above) using a Swimming Behavioural Recorder (SBR) experimental set-up. The SBR features a video camera with a macro-objective that records the paths of a sample of swimming larvae. The device is inserted in a black box (60 × 60 × 100 cm) to exclude external sources of light, and the recording chamber is monitored under infrared light. Swimming behaviour is digitally recorded for about 3 s at 25 frames/s, and images are analysed using advanced image processing software (SBR system developed by e-magine IT, Genoa, Italy). With this analysis, individual nauplius path-tracks can be reconstructed, and the average naupliar swimming speed (mm/sec) can be measured for each sample (15–20 organisms/1 mL well). Final data are obtained calculating the mean value of the six replicates (M ± ES; *n* = 6).

### Phase II: Toxicity bioassays with reference toxicant

In phase II experiments, ecotoxicological responses of *A. amphitrite* nauplii after different cold-storage times were assessed in the presence of Cadmium Nitrate. As described above for phase I, nauplii were transferred into multiwell plates, each well containing 1 ml of toxic compound solution at different concentrations (0; 0.2; 0.4; 0.8; 1.6; 3.2 mg/L) and stored at 20 °C in the dark. For cold storage trials, larvae kept at different (T1, T2, T3; see 2.5) cold-storage (4 ± 1 °C) times were used. As described in 2.5, about 15–20 organisms were placed into wells containing 1 ml of toxic compound solution at different concentrations (0; 0.2; 0.4; 0.8; 1.6; 3.2 mg/L). Three replicates were prepared for each concentration and the mean values of the three replicates (M ± ES; *n* = 3) were taken for controls and results. Mortality, immobilization, and swimming speed were recorded (as described in 2.5) after 24 h. As to behavioural end-point, data shown are referred to swimming speed alteration (SSA) normalised to the average swimming speed of the controls (0 mg/L) according to the formula: % Alteration (SSA) = [(S treated-S controls)/S controls]/100.

### Phase III: Toxicity bioassays with environmental samples

Responses obtained for *A. amphitrite* nauplii stored at 4 ± 1 °C before bioassay set up were observed after 24 h of exposure to a sediment elutriate. In this experimental phase, only two end-points (mortality and immobilization) were considered, since only acute toxicity response is specified in the legislation (D.M. 173/2016). As for phase II, for T0 (non-cold storage), nauplii were exposed to 1 ml of sediment elutriate, (100%), and stored at 20 °C in the dark. For cold storage trials, a bioassay was set up as described in 2.5 after different times (T1, T2, T3; see 2.5), at 4 ± 1 °C, by adding 15–20 organisms into wells containing 1 ml of elutriate. Three replicates were prepared for elutriate and for controls, while the results refer to the mean values of the three replicates (M ± ES; *n* = 3). Mortality and immobilization were recorded (as described in 2.5 and 2.6) after 24 h.

### Statistical analysis

All data are expressed as means ± standard error of the replicates. Acute toxicity tests were deemed valid if control immobility was less than 10% (USEPA, 2002). For phases II and III, Lethal Concentration (LC_50_: cadmium concentration or elutriate dilution resulting in 50% deaths of the exposed organisms after 24 h) and Effective Concentration (EC_50_: cadmium concentration or elutriate dilution resulting in 50% immobility or SSA effect in the exposed organisms after 24 h) and related 95% Confidence Limits (CL) were calculated using Spearman–Karber analysis (Finney 1978).

Significant differences between controls and treated samples were determined using one-way analysis of variance (ANOVA), followed by Tukey test. When data failed to meet the assumption of normality, the non-parametric Kruskal–Wallis test and Mann-Whitney test were used to compare individual treatments. For SSA test, statistical analysis was performed using swimming speed data. Data were considered significantly different when *p* < 0.05. SPSS statistical software (Statistical Package for the Social Sciences, Version 20) was used for data analysis.

## Results

### Phase I: Preliminary bioassays with clean natural sea water

**Fig. 1:**
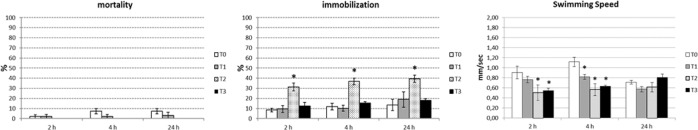
Mortality and immobilization percentages and Swimming Speed (mm/s) recorded after 2, 4 and 24 h of incubation in 0.22 µm clean FNSW at 20 °C (dark) for *A. amphitrite* nauplii stored at 4 ± 1 °C for 0, 24, 72 and 120 h (T0, T1, T2, T3) before assay set up (M ± ES; *n* = 6). Asterisk indicates = significant difference (*p < 0.05) from T0 (no cold storage)

Results of this experimental phase are reported in Fig. 1. Regarding mortality, after 24 h from bioassay set up, 7.3% of mortality was obtained for T0 nauplii, 3.15% for T1 nauplii, and no mortality was recorded both for T2 and T3 nauplii. Mortality percentages are always below 10% even for nauplii subjected to the highest storage time (T3, 120 h). Regarding immobilization, a significant difference in nauplii immobility can be observed only between T0 and T2 (72 h storage at 4 ± 1 °C) for all exposure times (after 2, 4 and 24 h). For the behavioural endpoint, the average speed of T0 (non-cold-stored organisms) and cold-stored nauplii (T1, T2, T3) recorded after 2, 4 and 24 h from assay set up is reported in Fig. 1. The average speed of nauplii recorded after 2 and 4 h decreases with increasing cold storage time (from T0 to T3), with significant (*p* < 0.05) differences between T0 and T2 and T3 when the end-point is recorded after 2 h, and between T0 and all cold storage times when speed is recorded after 4 h. Conversely, when the end point is recorded after 24 h from bioassay set up, no significant differences in swimming speed correlated with cold storage time are evident between T0 and cold storage time (T1, T2, T3).

### Phase II: Toxicity bioassays with reference toxicant

**Fig. 2:**
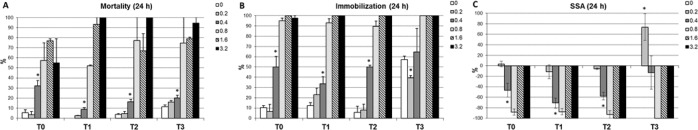
Mortality (**A**), Immobilization (**B**) and Swimming Speed Alteration (SSA) (**C**) percentages recorded after 24 h from bioassay set up with a Cadmium Nitrate solution (0; 0.2; 0.4; 0.8; 1.6; 3.2 mg/L) for *A. amphitrite* nauplii stored at 4 ± 1 °C for 0, 24, 72 and 120 h (T0, T1, T2, T3) before assay set up (M ± ES; *n* = 3). * = lowest cadmium concentration showing a significant (*p* < 0.05) difference from controls (0 mg/L)

Results of the second experimental phase are reported in Fig. 2. Data obtained for nauplii stored at 4 ± 1 °C for 0, 24, 72 and 120 h (T0, T1, T2, T3) before assay set up are reported. Responses observed after 24 h are shown, since this is the time indicated in the standardized protocol for end-point observation.

Mortality percentages in control organisms (0 mg/L of cadmium) are always ≤10% (Fig. 2A), even for nauplii exposed to the longest cold storage time (120 h, T3), thus meeting the acceptability criteria indicated in the standardized protocol for this bioassay (mortality percentage in control group <10% after 24 h). A significant difference from controls (0 mg/L) is recorded at all concentrations (from 0,4 mg/L up to 3,2 mg/L) for all cold storage times (see Fig. 2A). Immobilization for control group (0 mg/L) remains below 10% for T0, T1, and T2 (Fig. 2B), but it exceeds 50% for nauplii stored for 120 h (T3) at 4 ± 1 °C. A significant difference from controls (0 mg/L) is recorded at 0.4 mg/L for T1 and T2 organisms, while for T3 (120 h) a significant difference is already evident at the lowest cadmium concentration (0.2 mg/L; see Fig. 2B). For the behavioural end-point (Fig. 2C), naupliar swimming is inhibited by cadmium exposure at any concentration at all cold storage times. At 0.8 mg/L, swimming speed results to be inhibited by more than 80% than controls already at T0; significant (*p* < 0.05) speed alterations compared to controls are evident for T0, T1 and T2 from 0.4 mg/L, and already from 0.2 mg/L for T3. After 120 h (T3) of cold storage, swimming speed significantly increases at the lowest cadmium concentration (0.2 mg/L), while it is significantly inhibited at higher concentrations. This result can be ascribed to a hormetic effect (Calabrese and Baldwin 2001; Zamunda and Sunda 1982). For the other cold storage times (T0, T1 and T2) swimming speed inhibition is observed at all cadmium concentrations.

LC_50_ (for mortality) and EC_50_ (for immobilization and swimming speed alteration) were calculated for each cold-storage time (T0, T1, T2 and T3) and are reported in Table 1.

**Table 1:** LC_50_ (mortality) and EC_50_ (immobilization and Swimming Speed Alteration) after 24 h from bioassay set up with Cadmium Nitrate solution (0; 0.2; 0.4; 0.8; 1.6; 3.2 mg/L) for *A. amphitrite* nauplii stored at 4 ± 1 °C for 0, 24, 72 and 120 h (T0, T1, T2, T3) before assay set up

	LC_50_ 24 h (mg/L) *Mortality*	EC_50_ 24 h (mg/L) *Immobilization*	EC_50_ 24 h (mg/L) *SSA*
**T0**	0.75 (0.57–0.99)	0.43 (0.40–0.46)	0.44 (0.41–0.48)
**T1**	0.77 (0.70–0.85)	0.49 (0.45–0.54)	0.34 (0.31–0.37)
**T2**	0.78 (0.70–0.86)	0.44 (0.40–0.48)	0.38 (0.35–0.41)
**T3**	0.78 (0.70–0.86)	0.45 (0.42–0.48)	<0.2

LC_50_ after 24 h of Cadmium Nitrate exposure is within the 0.75–0.78 mg/range L, and confidence limits (95% CL) of each value include LC_50_ obtained for other storage times. As to immobilization, the EC_50_ range is wider (0.43–0.49 mg/L), but still within the acceptability range indicated in the standard protocol for this bioassay (NU 2245/2012), which is 0.2–1.3 mg/L (EC_50_ for immobilization after 24 h). For SSA, the EC_50_ range after 24 h shows the highest variability (0.2–0.44 mg/L) among the three considered end-points.

### Phase III: Toxicity bioassays with environmental samples

**Fig. 3:**
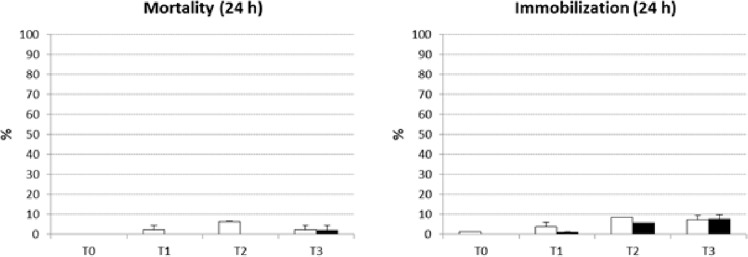
Mortality and immobilization percentages after 24 h of contact with the environmental matrix (sediment elutriate) for *A. amphitrite* nauplii stored at 4 ± 1 °C for 0, 24, 72 and 120 h (T0, T1, T2, T3) before assay set up (M ± ES; *n* = 3). Control water (clean 0.22 µm FNSW): white bars; Sediment elutriate: black bars

Results of the third experimental phase are reported in Fig. 3. Mortality and immobilization percentages were recorded after 24 h of *A. amphitrite* nauplii exposure to sediment elutriate. For both end-points, less than 10% effect was recorded, even for nauplii stored at 4 ± 1 °C for 120 h (T3).

## Discussion

The aim of this paper was to determine whether low temperature (4 ± 1 °C) storage of *A. amphitrite* nauplii before their use in bioassays could affect ecotoxicological responses. Information about sensitivity changes after cold storage for a further ecotoxicological application becomes very useful, in order to optimize efforts by laboratories in maintaining adults cultures. This study considered a maximum storage time of 5 days, which was deemed suitable both in order to decrease laboratory work necessary to obtain newly hatched larvae, as well as in the case of larvae supplied to other laboratories. The nauplii of the crustacean *A. amphitrite* have proved to be an excellent model organism for ecotoxicity tests (Faimali et al. 2005; Garaventa et al. 2010; Greco et al. 2006; Piazza et al. 2009, 2012) with high sensitivity to a wide range of toxicants, such as heavy metals, neurotoxic pesticides, antifouling biocides, microplastics and surfactants (Bressy et al. 2010; van Dam et al. 2016; Faimali et al.2006b; Gambardella et al. 2017; Greco et al. 2006; Piazza et al. 2011, 2018).

As reported in the introduction, cryopreservation of organisms is expensive and not suitable prior to ecotoxicological testing. Maintenance of larvae in the cold (2–6 °C) and dark is less expensive and more realistic and viable (Anthony et al. 1996; Lubzens et al. 1996), but still few data are available in the literature on the effects of cold storage on ecotoxicological end points. For the copepod *Acartia tonsa* hatching success of eggs after cold storage was investigated (Hagemann et al. 2016; Drillet et al. 2006), A. *tonsa* larval stages are used as live feeds in aquaculture, but they are also a model organism adopted in ecotoxicological studies. It has been shown that, after keeping eggs at 3 °C for up to 11 months, hatching rates higher than 70% can still be achieved (Drillet et al. 2006). However, it was observed that the copepod community originating from cold-stored eggs reported higher mortality than those originating from fresh eggs, thus indicating that a cold storage treatment is not suitable for this model organism when intended for use in ecotoxicological testing. Cold storage treatment was also tested for another model organism adopted in toxicological bioassays–oyster larval stages–but only for aquaculture purposes, not for ecotoxicological studies. Larvae of *C. gigas* were shown to be able to settle also after a 98-hour storage at 6 °C (Holiday et al. 1991).

As to barnacles, no studies can be found in the literature reporting data obtained in ecotoxicological tests after cold storage. The impact of temperature on different end-points (both acute and behavioural) has been observed, for example a correlation between temperature and swimming behaviour was found for stage VI nauplii of the acorn barnacle *Semibalanus balanoides* (Sorochan and Metaxas 2017). In this case, temperature was found to increase swimming persistence length. A No-Effect Range (NER) for temperature was found for *A. amphitrite* nauplii on different ecotoxicological responses (Piazza et al. 2016), including mortality, immobilization, and swimming speed alteration: as to mortality, NER was found at 5–30 °C. Within this range, organisms resulted not to be affected by temperature variation during the 24-hour exposure.

In general, very few cold treatments are applied to marine ecotoxicological studies, and only limited to cryopreservation (Paredes and Bellas 2009; Cantarino Ribeiro et al. 2018).

In this study, we have performed three experimental phases, each one considering potential cold-storage effects on naupliar ecotoxicological responses with and without the presence of a reference toxicant (Cadmium nitrate) or an environmental matrix (sediment elutriate), respectively. According to our results, mortality is a reliable end-point even after maximum storage time (5 days) considered.

Phase II experiments showed that mortality is the end point least affected by cold treatment: naupliar response does not change significantly at different storage times. The different sensitivity of considered end points is correlated with the variability range found for LC_50_ and EC_50_, calculated after 24 h from bioassay set up (Table 1). This range results to be very reduced for mortality, as LC_50_ calculated for T0, T1, T2 and T3 are always included in respective 95% confidence limits while for the behavioural response (swimming speed alteration) greater differences in EC_50_ values are recorded at different cold storage times. The reason for this result lies in physiological changes that could may occurr in the larvae after cold storage, determining different behavioural changes during end-points observation. As regards swimming speed, the larval swimming speed was higher for T3 cold-stored nauplii respect to the speed registered for nauplii subjected to shorter cold storage times (T1 and T2) or not cold stored (T0). In detail, T0 nauplii have (after 24 h from bioassay set up) an average speed of 0.71 mm/s, for T1 nauplii the average speed is 0.57 mm/s, for T2 is 0.61 mm/s and for T3 is 0.81 mm/s. Even small physiological changes (due to temperature) are able to alter naupliar behaviour, consequently to determine small variations in swimming speed.

The feasibility of using cold stored nauplii in environmental monitoring testing was investigated in Phase III experiments. The end-points were observed after 24 h. In this case too, mortality resulted to be a reliable response, with less than 10% effect and no significant differences in the results obtained with T0 and T1, T2 and T3 nauplii. This latter experimental phase is of great importance, as the acute toxicity assay with *A. amphitrite* larvae is required under Italian regulations regarding assessment of dredging sediment quality (D.M. 173/2016) with reference to the mortality end point (after 24 h). Being able to use cold-stored organisms (up to 5 days) would have a positive impact also in environmental monitoring, encouraging the use of this model organism.

## Conclusions

Data obtained with the three different experimental phases adopted in this study show that short (up to 5 days) cold storage (4 ± 1 °C) of *A. amphitrite* nauplii before bioassay set up does not seem to affect their acute ecotoxicological response, in particular, if the lethal end-point (mortality) is to be considered for final evaluation. This could offer an advantage in optimizing and reducing laboratory efforts to obtain newly hatched larvae from adults, thus raising the pool of model organisms to be used in ecotoxicological bioassays.

## Data Availability

The datasets used and/or analysed during the current study are available from the corresponding author on reasonable request.
